# Editor’s Book Table

**Published:** 1869-11

**Authors:** 


### Editor's Book Table.

[NOTE.—All works reviewed in the columns of THE CHICAGO MEDICAL JOURNAL may be found in the extensive stock of W. B. Keen & Cooke, whose catalogue of medical books can be had on application.]

*Sleep, and its Derangements.* By WILLIAM A. HAMMOND, M.D., Professor of Diseases of the Mind and Nervous System, etc., Bellevue Hospital Medical College. Philadelphia: J. B. Lippincott & Co. 1869. Pp. 318. W. B. Keen & Cooke.

PROF. HAMMOND is one of our most industrious physiologists and most acute observers. In the book before us he has brought together a variety of facts, experimental and observed, which render it both valuable and interesting. However we may dissent from some of his conclusions, he manifests a zeal for discovery, and a candor and clearness in stating his opinions, which entitle whatever he writes to a respectful consideration. We have read the present treatise throughout with pleasure, and, at times, we might almost say, with fascination, and yet have laid it down with a vivid sense of disappointment. Perhaps no essay on the subject could fail in this result; nevertheless we are convinced that a proper recognition by the author of certain well established physiological principles, would have served to clear up portions of his subject which are now left obscure. The commanding professional position, and worthily won wide reputation, of Prof. Hammond, demand more than a passing notice of what he puts on professional record.

After calling attention to the necessity of sleep, or cessation of action for a temporary period, for each organ or apparatus of the system, the heart resting six hours of the twenty-four, the lungs eight, etc., and that this necessity probably extends even to organizations of the vegetable world — wherever use causes decay — he proceeds to discuss the causes of sleep, and adduces the varied opinions which have obtained, the most referring it to congestion of the cerebral vessels, or pressure from some cause on the cerebral mass, he observes that this idea has prevailed from observing the *stupor* which notably results from these causes. But, according to Dr. H., stupor, coma and sleep are neither identical, nor even phases or grades of a similar condition. The only point of resemblance between coma and sleep consists in the fact that both are accompanied by a loss of volition. We quote:

1. In the first place, a stupor never occurs in the healthy individual, while sleep is a necessity of life.

2. It is easy to awaken a person from sleep, while it is often impossible to awaken him from stupor.

3. In sleep the mind may be active, in stupor it is, as it were, dead.

4. Pressure upon the brain, intense congestion of the vessels, the circulation of poisoned blood through its substance cause stupor, but do not induce sleep. For the production of the latter condition a diminished supply of blood to the brain, as will be shown hereafter, is necessary.

In investigating this last proposition, the author trephined dogs, and observed carefully, and even, by an ingenious apparatus described in the appendix, measured the collapse of the cerebral mass during sleep. Similar observations in cases of traumatic removal of the skull from the human being, congenital deficiency, the fontanelles of infants, etc., concurred to the same judgment.

"We thus see that the *immediate* cause of sleep is a diminution of the quantity of blood circulating in the vessels of the brain, and that the *exciting* cause of periodical and natural sleep is the necessity which exists that the loss of substance which the brain has undergone should be restored.

* * * "Whatever other cause is capable of lessening the quantity of blood in the brain is also capable of inducing sleep. There is no exception to this law, hence we are frequently able to produce this condition at will."

Thus heat, cold, voluntary diminution of attention, or fatigue, the process of digestion, excessive loss of blood, debility, and the action of certain medicines.

In the next chapter, describing the physical phenomena of sleep, it is stated that

"The ganglionic nervous system and the spinal cord continue in action during sleep, though generally with somewhat diminished power and sensibility. The reflex faculty of the latter organ is still maintained, and thus varied movements are executed without the consciousness of the brain being awakened.

"Somnambulism is clearly a condition of exaltation in the functions of the spinal cord, without the controlling influence of the cerebrum being brought into action."

The fourth chapter considers the state of the mind during sleep, with the following conclusions:

1. Feeling — embracing sensation and emotion — is suspended, so far as the first is concerned; but is in full action as regards the second. We do not see, hear, smell, taste or enjoy the sense of touch in sleep, although the brain may be aroused into activity, and we may awake through the excitations conveyed to it by the special senses. The emotions have full play, unrestrained by the will, and governed only by the imagination.

2. The will or volition is entirely suspended.

3. The thought or intellect is variously affected in its different powers. The imagination is active, and the memory may be exercised to a great extent; but the judgment, perception, conception, abstraction, and reason are weakened, and sometimes altogether lost.

The fifth chapter treats of the Physiology of Dreams. His opinion is summed up in the statement that

"Dreams are directly caused by an increased activity of the cerebral circulation over that which exists in profound sleep. This activity is probably sometimes local and at others general, and never equals that which prevails in the condition of wakefulness, when the functions of the brain are at their maximum energy."

In this chapter occurs the following paragraph, to which we wish to direct attention. We quote the sentences only to enter, also, our earnest dissent and protest. It is on a par with some of the teachings in poor old Paine's "Institutes," and is grossly unworthy the magnificent intellect of Prof. Hammond:

"Writers who contend for the doctrine of constant mental activity regard the brain as the organ or tool of the mind, a structure which the mind makes use of in order to manifest itself. Such a theory is certain to lead them into difficulties, and is contrary to all the teaching of physiology. The full discussion of this question would be out of place here; I will therefore only state that this work is written from the standpoint of regarding the mind as nothing more than the result of cerebral action. Just as a good liver secretes good bile, a good candle gives good light, and good coal a good fire, so does a good brain give a good mind. When the brain is quiescent there is no mind."

The sixth chapter treats of Morbid Dreams—prodromic, symptomatic and essential.

The seventh, of Somnambulism, in which he states (p. 218):

"Much observation and many experiments have convinced me that the importance of the spinal cord, as a centre of intellection and volition has been unwarrantably ignored.

"It is, of course, not a matter for doubt that the faculty of consciousness is latent in the spinal cord, so long as the brain is in a state of activity, and that the faculty of memory does not reside in it at all."

Sometimes in the progress of diseases the cord obtains the mastery of the superior organ, and dominates with terrible power. All sense exercised during somnambulism owes its activity to the spinal cord, and in most cases the brain is so profoundly asleep that no impressions whatever are transmitted to the cord, and therefore there are no sensations whatever except that of touch.

In artificial somnambulism—the hypnotism of Braid—this power of the cord is even more higly manifested, and the brain is still even more incapable of control.

The concluding chapters are devoted to the Pathology and Treatment of Wakefulness, Somnolence and "Sleep Drunkenness."

The limits of the present notice will not permit further extracts or even brief abstracts, as we should desire.

Without entering into any general discussion here, we beg leave to append these suggestions:

1. The greater or less amount of blood contained in the cerebral vessels, *per se* explains nothing. There is, when the bones are fully developed and united, a uniform pressure; when there is less blood in the vessels the cranial cavities and cerebral interspaces contain an increased amount of serum. When there is an increased amount of blood there is diminution of the cranio-spinal serum. Cessation of nutrition of the brain mass reveals, *post mortem*, "water on the brain." Hence abundant misconception of necroscopic observations. The amount of blood flowing *through* a part is determined by the amount of tissue metamorphosis, cell or molecular, taking place in that part, whether brain, muscle or gland. Diminished organic change in the tissue of the cerebral mass may lessen the amount of blood *in* it; but it does not follow that every diminution of the mass of blood will lessen the amount of organic change. More of the blood is used. This is the case often in exhausting hæmorrhages. Rapid organic change is still going on, involving extreme vigilance, and still further impoverishing the blood. Hyperæmia and congestion are not synonymous terms. The first may consist essentially in velocity of transit and not in massing up of the blood. In passive congestion there is slower transit or actual impaction of blood in the vessels. It is not the *pressure* here which lessens the activity of the vital manifestation — brain or other — it is the cessation, or at least diminution of organic change, a circumstance wholly irrelevant to pressure, except in so far as pressure may be a cause of the diminished organic change.

It seems to us nearly time to get our medical language freed from these hydraulic phrases, which have ceased to have any appropriateness or significance.

In sleep there is a diminution, *pro tanto*, or cessation of nutritive changes in the true nervous substance, although the phenomena suggest that the process of removal of effete matters is still going on.

It would appear, indeed, that this latter is an essential circumstance for the occurrence of sleep, for nothing more conduces to wakefulness than imperfect elimination. No agents are better adapted to secure sleep than those which energize this process. This is probably the *rationale* of the use of Bromide of Potassium in wakefulness — a remedy which Dr. H. especially lauds.

Those agents which, like opium and alcohol, in large doses directly check organic changes, are notably followed by unpleasant secondary effects unless activity of elimination is in some manner secured. Indirect narcotics are always therapeutically the best, unless perchance in acute inflammation or other causes of excessive tissue change.

In the stupor or coma from drugs, mechanical pressure, cold, etc., etc., there is simply a more or less complete cessation, both of organic nutrition and elimination; it is not merely a hydraulic process.

2. What *we* call the mind is an assemblage of faculties known to us by its manifestation through certain organized parts. It is not, in our view, a "secretion" any more than light is a secretion of the retina, or sound of the auditory nerve. Its force or forces we can as well conceive determining cell or molecular changes upon the vesicular expansions of neurine of the cerebral masses, as we can of light rearranging the retinal surface. When the eyelid is closed, we are unwilling to say there is no light, and when sleep stops the changes which in waking moments continually take place in vesicular neurine, we are equally unwilling to say, with Prof. Hammond (p. 111), "*there is no mind.*" We do not dissent from his statement from any mere dread of materialism, but simply because in our view the doctrine is unphilosophical in the extreme, without even the redeeming quality that in some way it tends to simplify and explain.

3. After the first impression of the causes of common, special or mental sensation upon the structure adapted to receive it, all subsequent manifestations are strictly automatic. The mind acts upon and through the nervous system by as truly automatic a mechanism as does the physical stimulus through parts connected through the spinal cord.

Failure to appreciate the phenomena of somnambulism, of whatever character (hypnotism, etc.), arises from want of understanding of this automatic action of the mental faculties, with or without consciousness. Just as some people conceive the excito-secretory process to be something essentially distinct from the excito-motory, so very many seem to apprehend that the mental stimulus acts somehow differently from other stimuli in determining the so-called *reflex* phenomena of life.

Professor Hammond recognizes the "unconscious cerebration" of Carpenter, and yet not coupling this with the true mechanism of automatic mental manifestations, is driven to conceive of a previously ignored centre of intellection, volition and consciousness in the spinal cord (p. 218) !

But we have already vastly surpassed the space we can allot to this book. Let our readers buy and read it, for it is well worth attentive study.

*A Handy-Book of Ophthalmic Surgery;* for the Use of Practitioners. By John Z. Laurence, F.R.C.S., Assisted by Robert C. Moone. Second Edition. Revised and Enlarged by J. Z. Laurence. Philadelphia: H. C. Lea. 1869. Pp. 227. W. B. Keen & Cooke.

Among the numerous works on this specialty the work of Laurence must take a high rank. It is concise, yet clear and practical. For the busy practitioner, who wishes to consult a high authority and yet not wade through the sea of technicalities which the majority of ophthalmologic treatises half drown us in, this manual is especially to be commended.

It is freely illustrated, and elucidates in a highly practical manner the methods of examination, pathology and curative management of all the leading affections of the eye which are likely to be met with in general practice.

*The Pathology and Treatment of Stricture of the Urethra and Urinary Fistulæ.* By Sir Henry Thompson, F.R.C.S., etc. From Third and Revised London Edition. Philadelphia: H. C. Lea. 1869. Pp. 359. W. B. Keen & Cooke.

Contrary to the usual practice of authors, especially when a book has proved its popularity by passing to a third edition, this author has cut down the bulk of his treatise upwards of eighty pages. Points once in controversy having been settled, need no prolongation of the discussion, and the matured convictions of a well-known competent observer do not demand a tedious catalogue of detailed illustrative cases. The Anatomy and Physiology of the Urethra, with the Pathology of the different varieties of Stricture, their causes, etc., are first given, and then succinct accounts of the most approved medicinal and surgical modes of treatment. It is, perhaps, the best monograph on the subject.

### Pamphlets.

*Surgery of the Cervix in connection with the Treatment of certain Uterine Diseases.* By THOMAS ADDIS EMMET, M.D., etc., New York City.

IN this monograph Dr. Emmet discusses several topics of interest. He justly thinks too much importance has been attached to a detected erosion or so-called ulceration of the neck, when the condition of the cervix is generally but an effect and not a cause.

In the condition known as endometritis, with flexure, he uses large vaginal injections of water at from 98° to 104°. General therapeutic measures, of course.

For local treatment he prefers a solution of chromic acid, Churchill's strong tinct. of iodine, the persulphate of iron, and various other astringents, p. r. n.

These he applies by means of his uterine probe or applicator, by which he can get the exact sweep of the canal. saving thereby much pain to his patient. A little cotton is wound around the applicator after the proper curve has been given it, this saturated with the medicament and carried to the fundus. The recumbent position is insisted upon until the effects of the application have passed off. Sponge tents may be required. Twice a month is as often as these applications should be made.

If, after a reasonable period, the flexure remains unchanged, division of the cervix may be justifiable. The important point here is to ascertain whether the flexure is above or below the vaginal junction. Above the junction, division should be considered exceptional to the rule — below this point, it is not infrequently of great service — both for the relief of dysmenorrhœa and sterility. Except in cases of constriction of the os following the use of nitrate of silver, or from other causes, or fibrous tumors, he now never incises the cervix laterally. A division should never be attempted when the existence of perimetritis is suspected.

After detailing the steps of the operation, he calls attention to the fact that alarming hæmorrhage may supervene some considerable time after the operation, from vessels which, when first divided, contracted so that there was very trifling bleeding. Careful plugging is necessary, and the cervical pledget should be allowed to come away by suppuration.

"Retroflexion can be cured by the long continued use of hot water injections and hot baths, blistering the neck occasionally to deplete by the watery discharge, daily glycerine dressing, and by a careful attention to the state of the bowels and general condition. The malposition may be gradually removed by frequent manipulations: the finger of one hand in the rectum lifting the fundus, and a finger of the other hand in the vagina depressing the cervix. Modifications of this plan by the use of atmospheric pressure, are sometimes useful.

Dr. E. faithfully exposes the disastrous results of improper incisions, and explains cases in which amputation of the cervix may be best.

Attention is directed to the fact that a certain amount of prolapsus may be present, so that without due caution (as has occasionally happened) the peritoneal cavity may be opened at a point apparently distant from the vaginal junction.

Many females, he states, have been assured of cure of the erosion, and yet left in a miserable condition. The neck is found smooth and hard, and, in fact, a mass of cicatricial tissue. Oftentimes the situation is analagous to the "irritable stump." These cases sometimes resist all local treatment, and require excision. It is, however, unnecessary to remove more than a thin section from the entire surface to a limit at which the surrounding tissue can be found free enough to be drawn over the stump, after the plan of Dr. Sims.

The successful result of local treatment is in inverse proportion to the remaining induration, for it is impossible for the uterus to properly perform its function if the cervix is left a mass of cicatricial tissue. As a surgeon, Dr. E. remarks, he has always been conservative, but he has been forced to have recourse to surgical measures, and acknowledges that his ingenuity has been oftener taxed to repair damages inflicted by means used to afford relief, than from the result of disease uncomplicated.

We sincerely hope that those who "think they have mastered the art as a specialty when once in possession of a porte-caustique and a speculum," will ponder these timely words of Dr. Emmet.

*Inter-Dental Splint for the Treatment of Fractures of the Inferior Maxilla.* By E. A. Clark, M.D., Resident Physician St. Louis City Hospital.

Dr. Clark has been devoting large attention to the improvement of surgical apparatus and dressings for several years past, and has introduced several valuable improvements. In fracture of the inferior maxilla he contrives two vulcanized gutta-percha splints, conforming accurately to the shape of the teeth in both jaws, or to the alveoli when the teeth are wanting. The upper and lower splints are associated by means of pivots and triangular springs, so that their force will press the inferior maxilla downwards, the splint itself preventing lateral motion. The rubber splints are modeled to the case in the same manner that the dentist prepares his artificial alveolus.

Although not mentioned by Dr. Clark, a similar mechanism is useful in cases of exsection of the lower maxillary. Many years since, the late Dr. Brainard devised such a splint, which was manufactured for him by the well-known dentist, Dr. W. W. Allport, of this city. The appliance in that case proved eminently satisfactory, although several other forms of dressing failed to retain the parts in position.

The great facility with which the vulcanized rubber may be prepared in any shape desired, suggest it as eminently deserving of more general use than has heretofore been made of it.

*Our Navy. Its Offence is Rank. Rank in the Navy. Petition. Circular.* From D. B., Surgeon U. S. N.

We had supposed, until quite recently, that a gradual amelioration had taken place in the usages in the naval medical service, which, many years ago, called forth the united remonstrances of the profession. It appears, indeed, that some steps in advance were taken by the government. We quote from one of the papers referred to above:

"The Honorable Gideon Welles, who, from his experience as Secretary of the Navy during the entire war, had good reason to know the value of the other branches of the Navy, established by Regulation, as had ever been the custom in the Navy, certain advancements in their (the staff's) rank, in the lower grades only, partially commensurate with that of the line officers, — not advancing them, however, in the higher grades. This was done after full consultation, and the unanimous concurrence of the Attorney-General, the entire Cabinet, including President Lincoln, and it is said, also, the whole Supreme Bench. The rank so conferred stood for years as a matter of history and fact. Officers were appointed and confirmed by the Senate, and commissioned as surgeons, paymasters, and engineers, with the rank of lieutenant-commander — a new grade in the navy — under the 'Regulations of the Navy,' which were patent to the world, and were recognized by Congress during several years as valid and in full force."

Nevertheless, on the accession (temporarily) of Mr. Secretary Borie, he decided, on the strength of the opinion of Attorney-General Hoar, that this entire action was illegal, thus remanding the promoted officers, without trial or charges, to the lower rank — *in statu quo ante bellum* — the war wherein they had distinguished themselves, as a class, certainly not less than the officers of the line.

"It so happens that the last Act of Congress on this subject was the indorsement of the General Order of Secretary Bancroft, of August 31, 1846, by Congress, on the 5th of August, 1854, which, according to the ruling of the present Secretary, appears to have been in force illegally for eight years. The effect of this old order, put into action at the present day by Secretary Borie, in his own Order, No. 120, is to stop the promotion of all staff officers at the grade of *commander*, and to reduce many surgeons and paymasters who are over forty years of age, and have been in the navy twenty-five years, to the grade of *lieutenant*. In 1846, that grade was equal to the present one of commander, and the order was a liberal one. Now, owing to the rapid promotion and increase of numbers in the line, a lieutenant in the navy, instead of being forty years of age, is a young man of twenty, who entered the navy during the war.

"To the lasting disgrace of the United States navy, let it be said, that it is the only service now existing where this aristocracy of the line is fostered and encouraged; all other navies have rid themselves of it years ago. In the French and Austrian navies, the staff officers are represented from the grade of rear admiral down to that of lieutenant; in the English and Spanish navies, from vice-admiral; while in the naval service of autocratic Russia, where there exists a superior title to admiral, that of general admiral, the senior medical officer is eligible to that rank, and the assistant surgeon rates as lieutenant, and not master, as with us, nor yet as ensign, as proposed by Mr. Grimes.

"The staff officers of the navy demand that they shall not be thus wantonly degraded; and at the next session of Congress will urge that honorable body to be put more nearly on a par, not only with the line branch of our own navy, but the naval organizations of the whole civilized world."

This demand does not seem to us unreasonable. In every particular of education, native ability and gentlemanly deportment, so far as our acquaintance and information extend, the medical staff "rank" the highest position they have ever been awarded or now claim. We trust not only that this claim may be granted, but such a degree of independence may be guaranteed, that hereafter, at least, no officer of the line may compel a wounded or sick man to go upon duty when the surgeon officially certifies to his inability. If the surgeon abuses his trust let him be tried for the offence, and a suitable penalty inflicted.

This, also, would seem a common-sense proposition; nevertheless, it is but a few weeks since a highly competent and conscientious surgeon was suspended, for two years, if we rightly remember, for refusing to send a disabled man upon duty, at the command of some jack-a-dandy officer of the line.

We know it is very easy to say to the naval surgeons, If you don't like your positions, resign them; but aside from personal considerations, of which we have not space here to speak, but which readily suggest themselves, it should be a matter of professional pride, as it most assuredly is of national and patriotic concern, that the navy should be able to secure and retain medical ability and experience of the highest grade.

*Steiger's Literarischer Monatsbericht.* A Catalogue of Books in the German Language, Imported, Published and for Sale by E. Steiger, Publisher and Bookseller, 22 and 24 Frankfort Street, New York. For September and October, 1869.

For those of our readers who wish to procure German and other foreign books and periodicals, we refer to this house as prompt and reliable.

*Lindsay & Blakiston's Publications.* Philadelphia, September, 1869. Catalogue, with prices.

*The History of four Cases of Chronic Inversion of the Uterus*, with the Account of an Operation Designed as a Substitute for Amputation. By T. Gaillard Thomas, M.D., Professor of Obstetrics, etc. New York.

An exceedingly important paper. A succinct account of the history, diagnosis, etc., of this serious difficulty is given, with detailed report of four cases; one by Thomas Addis Emmet, M.D., the second by J. Worster, M.D. (New York), the third and fourth by Dr. T. himself. In the last case, he performed the unique operation proposed as a substitute for amputation.

Conceiving the main obstacle to reduction to be a strictured condition of the involuted cervix or even permanent adhesions formed, he first attempted to relieve this by incision, fixing the point of constriction by the finger, then drawing the womb down and cutting through the tissue of the neck, as recommended by Aran. By this act an artery of considerable size was cut, and the hæmorrhage checked with extreme difficulty. Further efforts at taxis were abandoned.

A week afterward, Dr. T. made an incision in the abdominal wall, some two inches in length, as in the exploratory incision of ovariotomy, and having ascertained, by the finger inserted in the uterine sac, that there were no adhesions, he passed in a steel dilator, made on the principle of a glove stretcher, dilated the stricture, and afterwards readily restored the uterus to its place.

Speedy recovery ensued.

Several modifications in the operation are proposed for future cases, which space will not permit us to detail.

The operation was both bold and successful, and what is better, philosophical and promising.

